# Differential COX-2 induction by viral and bacterial PAMPs: Consequences for cytokine and interferon responses and implications for anti-viral COX-2 directed therapies^☆^

**DOI:** 10.1016/j.bbrc.2013.07.006

**Published:** 2013-08-23

**Authors:** Nicholas S. Kirkby, Anne K. Zaiss, William R. Wright, Jing Jiao, Melissa V. Chan, Timothy D. Warner, Harvey R. Herschman, Jane A. Mitchell

**Affiliations:** a National Heart & Lung Institute, Imperial College London, UK; b William Harvey Research Institute, Barts & the London School of Medicine, Queen Mary University of London, UK; c Department of Molecular and Medical Pharmacology, University of California Los Angeles, USA

**Keywords:** Cyclooxygenase, LPS, Poly(I:C), Toll-like receptor, TLR3, TLR4

## Abstract

Cyclooxygenase 2 (COX)-2 is induced by bacterial and viral infections and has complex, poorly understood roles in anti-pathogen immunity. Here, we use a knock-in luciferase reporter model to image *Cox2* expression across a range of tissues in mice following treatment with the either the prototypical bacterial pathogen-associated molecular pattern (PAMP), LPS, which activates Toll-like receptor (TLR)4, or with poly(I:C), a viral PAMP, which activates TLR3. LPS induced *Cox2* expression in all tissues examined. In contrast, poly(I:C) elicited a milder response, limited to a subset of tissues. A panel of cytokines and interferons was measured in plasma of wild-type, *Cox1^−/−^* and *Cox2^−/−^* mice treated with LPS, poly(I:C), MALP2 (TLR2/6), Pam3CSK4 (TLR2/1), R-848 (TLR7/8) or CpG ODN (TLR9), to establish whether/how each COX isoform modulates specific PAMP/TLR responses. Only LPS induced notable loss of condition in mice (inactivity, hunching, piloerection). However, all TLR agonists produced cytokine responses, many of which were modulated in specific fashions by *Cox1* or *Cox2* gene deletion. Notably we observed opposing effects of *Cox2* gene deletion on the responses to the bacterial PAMP, LPS, and the viral PAMP, poly(I:C), consistent with the differing abilities of the PAMPs to induce *Cox2* expression. *Cox2* gene deletion limited the plasma IL-1β and interferon-γ responses and hypothermia produced by LPS. In contrast, in response to poly(I:C), *Cox2^−/−^* mice exhibited enhanced plasma interferon (IFNα,β,γ,λ) and related cytokine responses (IP-10, IL-12). These observations suggest that a COX-2 selective inhibitor, given early in infection, may enhance and/or prolong endogenous interferon responses, and thereby increase anti-viral immunity.

## Highlights

• We report interactions of Toll-like receptors (TLRs) with COX enzymes *in vivo*.
• COX-2 was broadly induced by LPS (TLR4) but more locally by poly(I:C) (TLR3).
• COX-1/2 deletion modified the response to TLR activation in a TLR-specific manner.
• COX-2 deletion enhanced interferon responses to viral-type TLR3/7/9 ligands.
• COX-2 inhibition could provide a novel anti-viral therapeutic strategy.

## 1 Introduction

Cyclooxygenase (COX) enzymes catalyze the two-step conversion of arachidonic acid to the unstable prostaglandin (PG) intermediate PGH_2_, which is then further converted to a range of prostanoid mediators that include PGE_2_, prostacyclin (PGI_2_) and thromboxane (TXA_2_). Two COX isoforms, which catalyze identical reactions, exist. COX-1 is constitutively expressed in many tissues, and generally plays a role in homeostatic function [1,2]. In contrast, COX-2 is generally not expressed in most healthy tissues but is rapidly induced in response to mitogens [3] and cytokines [4] and is often present in elevated levels at sites of inflammation. COX-2 produces prostanoids that contribute to vasodilation, increased vascular permeability, leukocyte chemotaxis, fever and that potentiate nociception. Indeed, COX-2 is the target of both traditional non-steroidal anti-inflammatory drugs (NSAIDs) such as aspirin and ibuprofen, which inhibit both COX-1 and COX-2, and of newer COX-2 selective inhibitors such as celecoxib. These agents are widely used for the symptomatic control of pain and inflammation, particularly in patients with arthritis, reflecting COX-2 induction in the inflamed arthritic joint.

In addition to its expression in sterile inflammation, it is clear that COX-2 is induced by pathogens. For example, gram-negative bacteria, or lipopolysaccharide (LPS), which activates the prototypical bacterial pattern recognition receptor Toll-like receptor (TLR)4, can induce COX-2 expression both in isolated cells [5,6] and *in vivo* [5,7]. Viruses or viral mimetics such as poly(I:C) can also induce COX-2 in cultured cells [8–10]. However, no systematic comparison of the ability of bacterial and viral pathogen-associated molecular patterns (PAMPs) to induce COX-2 in different tissues has been described. Moreover, whilst prostaglandins are recognized as important modulators of the immune system [11], there is no study that compares the roles of COX-1 versus COX-2 in inflammatory responses induced by viral and bacterial PAMPs. Here, we use a “knock-in” *Cox2* luciferase reporter mouse[7], in which luciferase activity expressed from the endogenous *Cox2* promoter reflects *Cox2* expression, to both visualize and quantify the tissue distribution of LPS- and poly(I:C)-induced *Cox2* gene expression. We then explore the role of COX-1- and COX-2-derived prostanoids in modulating the inflammatory response to specific PAMPs, using mice deficient in either COX isoform.

## 2 Methods

### 2.1 Animals

*Cox1^−/−^* [1], *Cox2^−/−^* [12] and *Cox2^fLuc/+^* mice [7] were back-crossed onto a C57Bl/6J background and identified by genomic PCR. Experiments were performed on 10–12 week old male and female mice. Animal procedures were conducted in accordance with Animals (Scientific Procedures) Act 1986 and after local ethical review by the Imperial College Ethical Review Panel or the UCLA Animal Research Committee, as appropriate for individual experiments.

### 2.2 Bioluminescent imaging

*Cox2^fLuc/+^* mice were injected with poly(I:C) (8 mg/kg; i.p.; Sigma, UK), LPS (from *Escherichia coli* serotype 055:B5; 0.1 or 10 mg/kg; i.p.; Sigma, UK) or vehicle (saline) under brief isoflurane anesthesia. 4 h later, d-luciferin (125 mg/kg; i.p.; Xenogen, USA) was administered. After a further 15 min, the dorsal skin was shaved and bioluminescent emission from this area was recorded over 3 min, using an IVIS imaging system (Xenogen, USA). Animals were then euthanized by isoflurane overdose and tissues were rapidly dissected and arranged on cell culture dishes, following which luciferase activity was imaged *ex vivo*. In both cases, collected photon number and images were analyzed using Living Image software (Perkin Elmer, USA) and quantified as the peak photon release/pixel detected from each tissue.

### 2.3 Luciferase activity assays

After bioluminescent imaging, tissues were snap frozen for biochemical measurement of luciferase activity. Tissues were dissociated using a Precellys24 bead homogenizer and the supernatants loaded into white 96 well microtitre plates. The luminescence was then read after injection of Luciferase Assay Reagent (Promega, UK). Protein concentration of homogenates was determined using the bicinchoninic acid method (Perbio, UK) and luciferase activity was normalized for protein concentration.

### 2.4 Animal condition, body temperature and blood counts

Ligands to TLR2/1 (Pam3CSK4; 2 mg/kg; Invivogen, UK), TLR2/6 (MALP2; 60 μg/kg; Enzo Lifesciences, UK), TLR3 (poly(I:C); 8 mg/kg), TLR4 (LPS; 0.1 or 10 mg/kg), TLR7 (R848; 2 mg/kg; Enzo Lifesciences, UK), TLR9 (CpG ODN 1826; 2 mg/kg; Invivogen, UK) or vehicle (saline) were administered to wild-type, *Cox1^−/−^* and *Cox2^−/−^* mice by intraperitoneal injection, under brief isoflurane anesthesia. Immediately prior to injection, core body temperature was measured using a rectal thermometer. After 4 h, the gross physical condition of mice was scored (0–5) by two independent observers based on coat condition (e.g. piloerection), behavior (e.g. activity, response to investigator) and posture (e.g. hunching). Mice were then euthanized by CO_2_ narcosis, and body temperature immediately measured. Blood was collected, from the vena cava, into heparin (10 U/ml final; Leo Laboratories, UK). Whole blood cells counts were obtained using a commercial veterinary biochemistry service (IDEXX Laboratories, UK) and plasma separated from the remainder by centrifugation for cytokine analysis.

### 2.5 Plasma cytokine levels

Plasma levels of IL-1β, IL-4, IL-5, IL-10, IL-12, IFNγ, TNFα and KC were determined using a multiplex immunoassay system (Meso Scale Diagnostics, USA). Plasma levels of IP-10 and IFNλ (R&D Systems, USA) and IFNα and IFNβ (PBL InterferonSource, USA) were determined by individual ELISAs.

### 2.6 Isolated blood assays

Untreated wild-type, *Cox1^−/−^* and *Cox2^−/−^* mice were euthanized by CO_2_ narcosis and whole blood collected as above then treated with ligands to TLR2/1 (Pam3CSK4; 1 μg/ml), TLR2/6 (FSL-1; 1 μg/ml; Invivogen, UK). TLR3 (poly(I:C); 10 μg/ml), TLR4 (LPS; 1 μg/ml) or TLR7 (imiquimod; 10 μg/ml; Invivogen, UK), the NOD1 ligand C12-iE-DAP (1 μg/ml), mouse IL-1β (10 ng/ml) or saline. Treated blood was incubated for 24 h at 37 °C, after which the plasma fraction was separated by centrifugation and IP-10 and KC levels determined.

### 2.7 Statistics and data analysis

Data were analyzed using Prism 5.01 (GraphPad software, USA). Statistical significance was determined by two-way ANOVA with Dunnett’s post-hoc test unless otherwise stated, and data sets considered different if *p* < 0.05. Each *n* value represents a data point from a separate animal.

## 3 Results

### 3.1 *Cox2* gene induction by prototypical viral and bacterial PAMPs

**Fig. 1:**
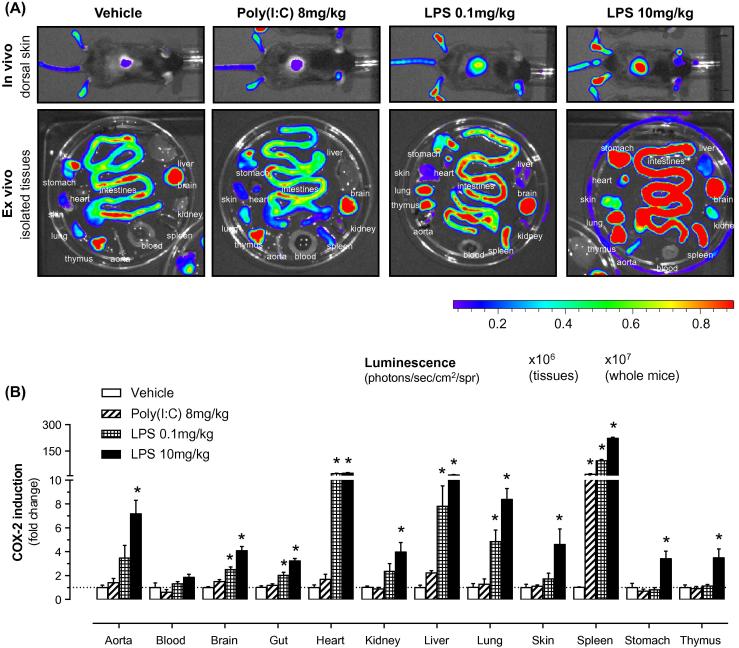
Expression from the *Cox2* gene induced in mice by prototypical bacterial and viral PAMPs. *Cox2* promoter-driven gene expression was measured, by bioluminescent imaging of skin *in vivo* and isolated tissues *ex vivo*, in *Cox2^fLuc/+^* luciferase reporter mice (A) and quantified as the fold-change in luminescence (B). The bacterial PAMP, LPS, produced a dose-dependent induction of *Cox2* gene expression across a broad range of tissues. The viral PAMP, poly(I:C), in contrast, had little effect on *Cox2*-driven gene expression in most tissues, but produced a selective *Cox2* induction in the spleen. Data are expressed as means ± s.e.m. from *n* = 4 to 5 individual animals per treatment. ^∗^*p* < 0.05 vs. vehicle.

*Cox2* gene expression is increased globally in *Cox2^fLuc/+^* animals treated with LPS, a TLR4 ligand [7]. In the current study, we extended our earlier observations and used two doses of LPS to observe a graded, dose-dependent increase in gene expression (Fig. 1A). When surface luminescence values for intact tissues were measured, the following tissues displayed a robust response (⩾2-fold increase versus vehicle-treated mice) to LPS (0.1 mg/kg): aorta, heart, liver, lung and spleen (Fig. 1A). When a larger LPS dose (10 mg/kg) was administered, all studied tissues measured displayed ⩾2-fold increase in *Cox2* gene expression (versus vehicle) with the following rank order spleen ≫ heart ⩾ liver > lung ⩾ aorta > skin ≈ kidney ≈ stomach ≈ thymus ≈ gut ≈ brain. In contrast to results obtained with LPS, the viral PAMP/TLR3 ligand, poly(I:C), at a dose (8 mg/kg), close to the maximum tolerated dose [13], produced only a modest induction of *Cox2* gene expression; significant induction was only apparent in the spleen (Fig. 1B).

Surface luminescence values do not always reflect the tissue activity in this model, since tissue absorbance of emitted bioluminescence and penetration of substrate *in vivo* influence measured luminescence. For this reason, we also measured luciferase activity, driven from the *Cox2* promoter, in tissue homogenates. These data (Supplementary Fig. 1) confirm those obtained by surface luminescence measurements and also reveal the stomach as a site of modest poly(I:C)-induced *Cox2* induction.

### 3.2 Modulatory role of COX-2 on physical features of sepsis induced by LPS *in vivo*

**Fig. 2:**
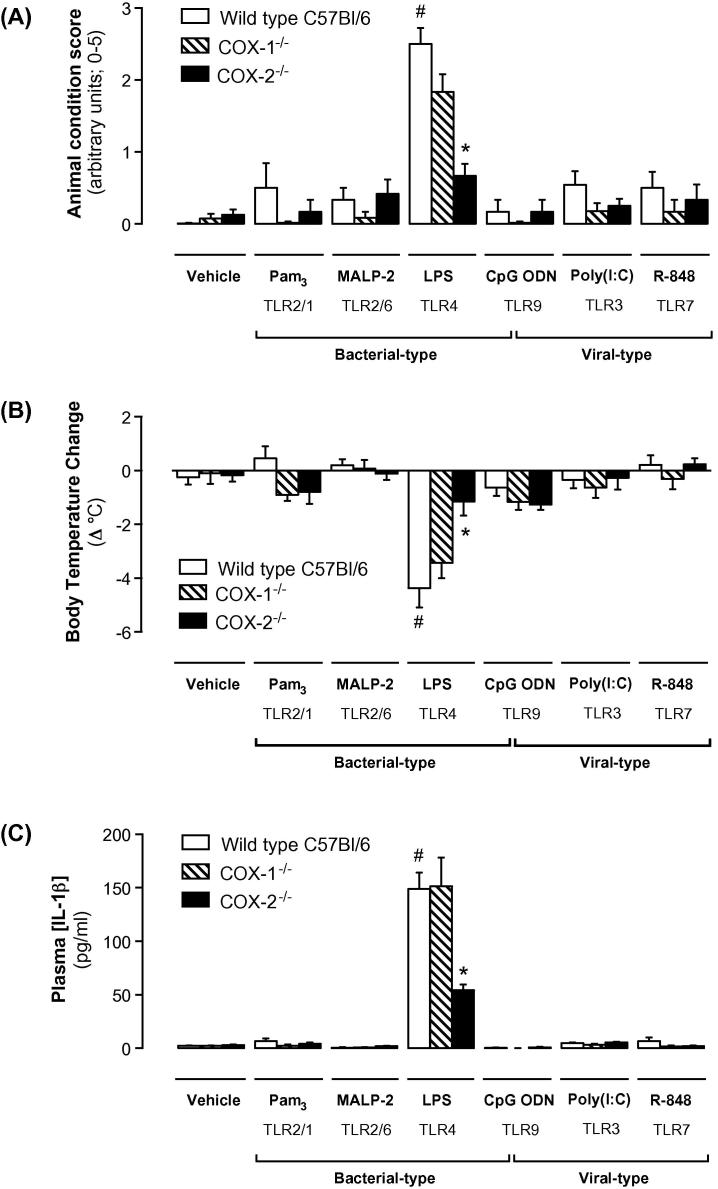
Effect of *Cox1* and *Cox2* gene deletions on the physiology of mice treated with bacterial and viral PAMPs. Wild-type, *Cox1^−/−^* and *Cox2^−/−^* mice were treated with a range of bacterial and viral PAMPs. After 4 h “animal condition” was scored (A), and core body temperature change (B) and plasma IL-1β levels measured (C). Only LPS produced an apparent of loss of condition in mice; this phenotype was accompanied by a hypothermic response and increased plasma IL-1β levels. *Cox2* gene deletion protected mice against each of these responses. Data are expressed as means ± s.e.m. from *n* = 6 to 12 individual animals per treatment. ^∗^*p* < 0.05 vs. wild-type; ^#^*p* < 0.05 vs. vehicle-treated wild-type.

Wild-type mice treated with LPS displayed observable *‘physical condition’* responses, including hunching, inactivity and piloerection (Fig. 2A). The physical condition response to LPS was accompanied by a decrease in body core temperature, reflecting loss of thermoregulation (Fig. 2B), and reduced circulating lymphocyte and platelet counts (versus vehicle treatment), suggesting tissue lymphocyte sequestration and intravascular coagulopathy, respectively (Supplementary Fig. 2). These LPS-induced behavioral and body temperature changes were COX-2-dependent, but not COX-1 dependent, since they were strongly suppressed in *Cox2^−/−^*, but not *Cox1^−/−^* mice (Fig. 2A and B).

In contrast to the results for LPS, other PAMPs tested did not affect the gross physical condition or body core temperature of the mice (Fig. 2A and B). Nonetheless, like LPS, Pam3CSK4, poly(I:C) and R-848 produced lymphocytopenia (Supplementary Fig. 2), whilst MALP-2, poly(I:C), R-848 and CpG ODN produced an increase in circulating neutrophils, probably reflecting mobilization from the bone marrow (Supplementary Fig. 2). *Cox1* or *Cox2* deletion did not affect any of these features.

### 3.3 PAMPs produce specific patterns of cytokine and interferon response *in vivo*

**Fig. 3:**
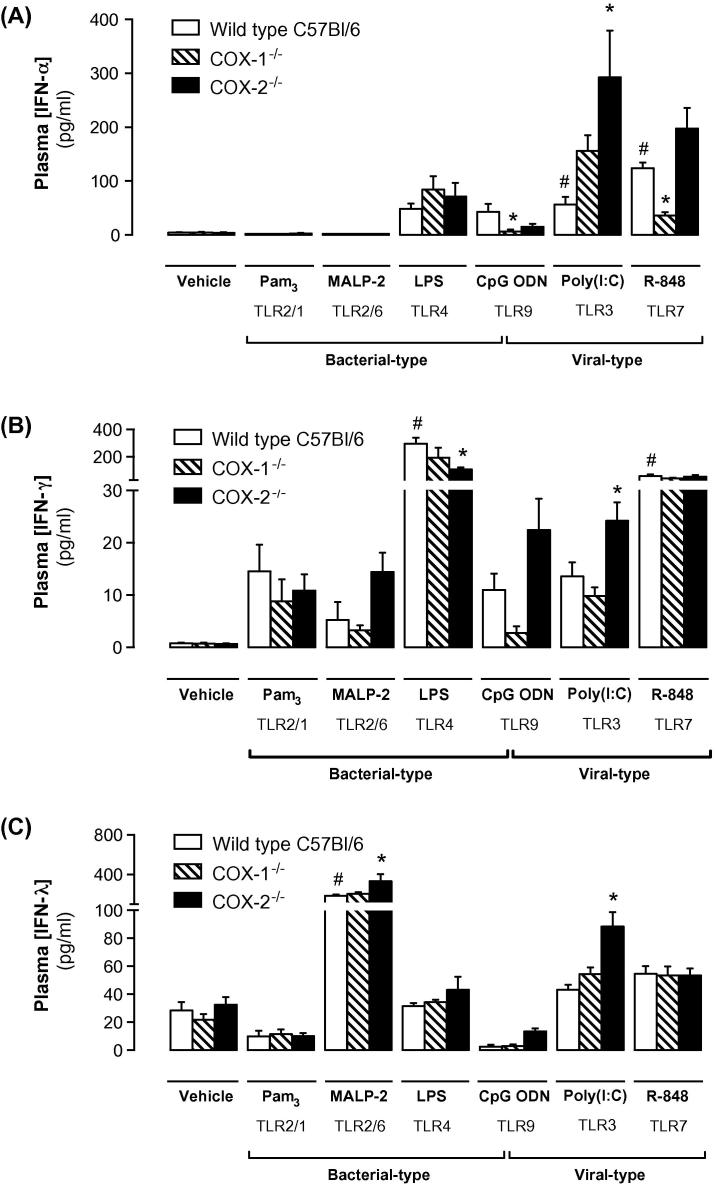
Effect of *Cox1* and *Cox2* gene deletions on circulating interferon levels in mice treated with bacterial and viral PAMPs. Wild-type, *Cox1^−/−^* and *Cox2^−/−^* mice were treated with a range of bacterial and viral PAMPs. After 4 h, the levels of IFNα (A), IFNγ (B) and IFNλ (C) were measured in plasma. In general, the viral PAMPs, poly(I:C), R-848 and CpG ODN, were better able to stimulate interferon production than their bacterial counterparts. In wild-type mice, poly(I:C) increased IFNα levels and tended to increase IFNγ levels, however, in each case the response was markedly increased in *Cox2^−/−^* mice. A similar pattern was true for poly(I:C)-induced IFNλ. In contrast, the IFNγ response to LPS was suppressed by *Cox2* deletion, whilst *Cox1* gene deletion limited the IFNα response to R-848 and CpG ODN. Data are expressed as means ± s.e.m. from *n* = 6 to 12 individual animals per treatment. ^∗^*p* < 0.05 vs. wild-type; ^#^*p* < 0.05 vs. vehicle-treated wild-type.

Each tested PAMP produced a specific pattern of plasma cytokine response, in agreement with reports from previous *in vivo* studies [14] and reflecting the distinct tissue distribution and signaling pathways of each TLR [15]. The pro-inflammatory cytokines IL-1β (Fig. 2C), TNFα and KC and the anti-inflammatory cytokine IL-10 (Supplementary Fig. 3) were increased in plasma of mice treated with LPS, but not in mice treated with the other PAMPs. IL-5 was also increased by LPS, as well as Pam3CSK4, whilst plasma IL-4 was elevated in mice treatedwith Pam3CSK4, CpG ODN and R-848 (Supplementary Fig. 3). Type I IFNs are intrinsically associated with viral infection [15]. Therefore, as expected, plasma IFNα levels were increased in mice treated with the viral PAMPs poly(I:C) and R-848. IFNα levels also tended to be increased in mice treated with the bacterial PAMPs LPS and CpG ODN (Fig. 3A). In wild-type mice, LPS and R-848 produced a significant increase in plasma IFNγ levels (Fig. 3B), but only MALP-2 produced a strong increase in the type III interferon, IFNλ (Fig. 3C).

### 3.4 Complex role of COX-2 in cytokine and interferon responses to viral and bacterial PAMPs

To understand how prostanoids produced by COX-1 and COX-2 modify the cytokine responses to specific viral and bacterial PAMPs, we performed studies using mice deficient in either isoform. In line with what was seen with physical condition and body temperature, in mice-treated with LPS, the IL-1β (Fig. 2C) IL-5 (Supplementary Fig. 3) and IFNγ responses were markedly suppressed by *Cox2* gene deletion (Fig. 3B; wild-type + LPS: 296 ± 43 pg/ml; *Cox2^−/−^* + LPS: 109 ± 13 pg/ml). By contrast, deletion of *Cox1* did not alter the cytokine response to LPS, perhaps reflecting an overwhelming *Cox2* induction by this stimulus (Fig. 1). More modest and variable roles for COX enzymes were noted in cytokine responses to other bacterial PAMPs, with effects of *Cox1* and *Cox2* deletion noted on specific IL-5 (Supplementary Fig. 3), IFNλ (Fig. 3) and IFNβ responses (Supplementary Fig. 4).

**Fig. 4:**
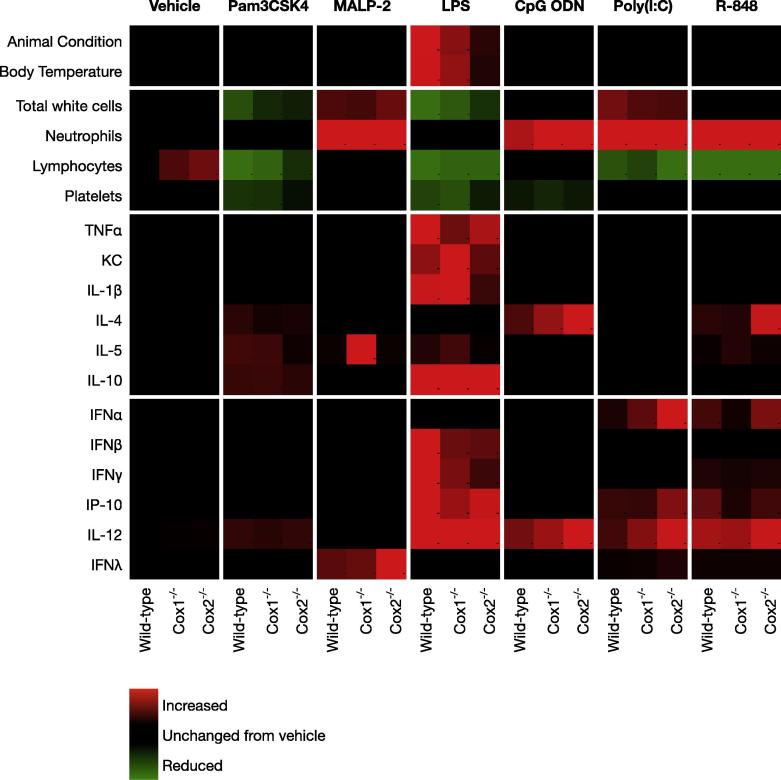
Heatmap summarizing the effect of *Cox1* and *Cox2* gene deletions on the physiology and cytokine response of mice treated with bacterial and viral PAMPs. Wild-type, *Cox1^−/−^* and *Cox2^−/−^* mice were treated with a range of bacterial and viral PAMPs. After 4 h animal condition, circulating blood cell counts and plasma cytokine levels measured. Data are presented as mean (*n* = 6–12) fold-changes from wild-type vehicle. For clarity, color change is only shown for parameters differing significantly from vehicle. Because ranges of change differ greatly for different ligands and/or responses, scale bars are not shown and individual columns/rows are normalized internally.

Cytokine responses to viral PAMPs exhibited a distinctly different pattern of response to *Cox1* and *Cox2* deletion. The most pronounced interactions was an *increase* of upto 6-fold in the IFNα, IFNγ and IFNλ (Fig. 4) and IFNβ (Supplementary Fig. 4) responses to poly(I:C) in *Cox2^−/−^* mice. This suggests that COX-2-derived prostanoids act to *limit* TLR3-mediated IFN release, and contrasts sharply with the pro-inflammatory role of COX-2 in LPS cytokine responses. In agreement, levels of the IFN-associated cytokine IL-12, and the IFN response cytokine IP-10 were also increased by *Cox2* deletion in poly(I:C)-treated mice, as were basal levels of IL-12 in vehicle-treated animals (Supplementary Fig. 4). Whilst there was a tendency for IFNα to be increased in poly(I:C) treated *Cox1^−/−^* mice, this effect was less robust than that seen in *Cox2^−/−^* mice and was in opposition to reduced R-848 and CpG ODN IFNα responses in *Cox1^−/−^* mice (Fig. 3). This further illustrates the specificity of the interactions between COX-1/2 activity and PAMP/TLR pathways. These effects of *Cox1/2* gene deletion on IFNs and related cytokines were associated with a systemic effect, since no change in IP-10 was noted in whole blood stimulated with PAMPs in culture. This contrasts to the clear increase in KC seen in whole blood stimulated with LPS (Supplementary Fig. 5).

## 4 Discussion

COX-2 is an inducible enzyme that regulates the production of prostaglandins in inflammation and infection. It is increasingly recognized that COX-2 has a complex role in immune responses, the extent of which we do not yet fully understand. In the current study we used a mouse model in which expression from the *Cox2* gene can be imaged directly, using a luciferase reporter “knocked in” to the coding region of the endogenous *Cox2* gene, to compare expression after treatment with bacterial and viral PAMPs. We present data showing that bacterial LPS caused a pan-COX-2 induction across all studied tissues. In contrast, the viral PAMP, poly(I:C), induced a much more tissue-specific and limited induction from the *Cox2* gene, with increases seen only in the spleen and stomach. These results likely reflect the limited distribution of TLR3 to specific immune cells, compared to broadly expressed TLR4.

In the current study, we show, for the first time, how deletion of *Cox1* or *Cox2* affects responses to a broad range of PAMPs. These data, summarized qualitatively in Fig. 4, clearly highlight distinct roles of COX-1 and COX-2-derived prostanoids in the modulation of specific PAMP/cytokine responses and suggest a potentially important role for COX-2 in anti-viral interferon responses.

In mouse models, the TLR4 ligand, LPS, induces a systemic inflammatory response that includes pronounced changes in the physical condition of the animal. Typical responses include piloerection, inactivity and loss of thermoregulation. In our study these effects were prevented by *Cox2* gene deletion, consistent with previous reports [16], but not by *Cox1* deletion. Importantly, these observations reflect what we know about the consequences of COX-2 inhibition in man; NSAIDs, including COX-2 selective inhibitors, are effective anti-pyretic drugs [17].

Whilst the other tested PAMPs did not induce notable changes in physical condition or thermal deregulation, they did produce cytokine changes. These effects were not ubiquitous and each PAMP elicited a relatively specific cytokine response profile (Fig. 4). *Cox1* and *Cox2* gene deletion studies suggested distinct interactions between COX-1, COX-2 and specific TLR responses. These differences likely reflect both the targeting of unique cell populations by each PAMP and the specific prostanoid pathways associated with these targets. Consistent with the anti-inflammatory effects of COX-2 inhibitors in man and in previous animal studies [16] we noted that *Cox2* gene deletion attenuated IL-1β, IL-5 and IFNγ responses to LPS. In contrast, the response to other tested bacterial PAMPs were, for the most part, not consistently altered by deletion of either the *Cox1* or *Cox2* gene.

TLR3, the receptor for poly(I:C) is highly expressed on dendritic cells, which secrete IFNα and IL-12. These ligands, in turn, stimulate IFNγ production in natural killer cells [14]. Interestingly, *Cox2* gene deletion enhanced poly(I:C)-induced release of type I (IFNα, IFNβ), type II (IFNγ) interferons and IL-12, consistent with an augmented dendritic cell response, as well as release of type III interferon (IFNλ) and the IFN response protein, IP-10. Indeed, elevated basal IL-12 levels in *Cox2^−/−^* mice suggest dendritic cells may be primed for activation in these animals, consistent with previous reports that prostanoids can limit dendritic cell function and survival [18]. TLR7 and TLR9, the receptors for the viral PAMPs R-848 and CpG ODN, respectively, have a broader expression in the immune system than TLR3 and couple to the MyD88 rather than TRIF adapter protein. Unlike TLR3-mediated IFNα responses, TLR7/TLR9 stimulated-release of IFNα was not enhanced by *Cox2* gene deletion and was suppressed by *Cox1* gene deletion, illustrating a complex interaction between viral PAMPs and the two COX enzymes.

In addition to the predicted anti-inflammatory role of COX-2 deletion, our observations showing enhanced anti-viral interferon responses in *Cox2^−/−^* mice to poly(I:C), suggesting that COX-2 inhibitors could have disease-modifying activity in viral infections. Interactions between COX-2 and viral infection has been suggested in a limited number of studies; COX-2 can be induced by whole virus in isolated cells [8–10] and COX-2 protein is increased in biopsy specimens of viral target tissues from patients with active viral infection [19,20]. Moreover, data from isolated cells [8,10] and animal models suggest that COX-derived products may play an important role in the host response. *Cox2* deletion reduces mortality in mice infected with influenza A [21]. Moreover, in the same model, *Cox1* deletion is associated with worsening of infection, consistent with our data demonstrating that COX-1 can limit TLR7/9 interferon responses. Protective effects of COX-2 inhibition have also been described in rodent models of respiratory syncytial virus [22] and vesicular stomatosis virus (VSV) [23]. Whilst there is little consensus as to the mechanism(s) responsible for this effect, limited previous data point to a role for IFN. For example, COX-2 inhibition in VSV-infected mice increases plasma IFNγ and IL-12 levels [23], and treatment of hepatitis B or C patients with the COX-1/COX-2 inhibitor indomethacin increases serum levels of the IFN response product 2′5′-oligoadenylate synthetase-1 [24]. Our data may, therefore, provide a mechanistic link by which COX-2 regulates viral infection as a result of modulation of innate immune recognition and subsequent interferon response.

Taken together, this study is the first to provide a systematic analysis of the reciprocal interactions between COX enzyme induction and inflammatory responses to a range of bacterial- and viral-like stimuli. The ability of *Cox2* deletion to suppress physical and cytokine responses to LPS has been previously reported, and contrasts strongly with our new data showing that *Cox2* deletion enhances IFN responses to viral PAMPs, particularly poly(I:C). These data suggest a role for COX-2 in limiting the anti-viral cytokine/interferon response to infection, and may provide a plausible explanation for the previously published data showing that *Cox2* deletion/COX-2 inhibition is beneficial in animal models of viral infection. If a similar mechanism is present in man, COX-2 inhibitors might be a potential anti-viral therapy, able to boost the endogenous anti-viral response when given soon after infection.

## Role of funding sources

This research was supported by a Wellcome Trust program grant (0852551Z108/Z; J.A.M.) and NIH-NCI P50 award CA086306 (H.R.H.). A.K.Z. is the recipient of an American Society of Hematology Scholar Award. No funding source was involved in the study design; data collection, analysis or interpretation; writing of the report or the decision to submit for publication.

## Appendix A Supplementary data

Supplementary data 1 This article contains supplementary material.
