# Supplementary material for: Differential COX-2 induction by viral and bacterial PAMPs: Consequences for cytokine and interferon responses and implications for anti-viral COX-2 directed therapies
Source: Biochem Biophys Res Commun. 2013 Aug 23;438(2):249–56. doi: 10.1016/j.bbrc.2013.07.006 (PMC3759847; doi:10.1016/j.bbrc.2013.07.006)
Supplement: Supplementary data 1 — This article contains supplementary material. [file mmc1.pdf]

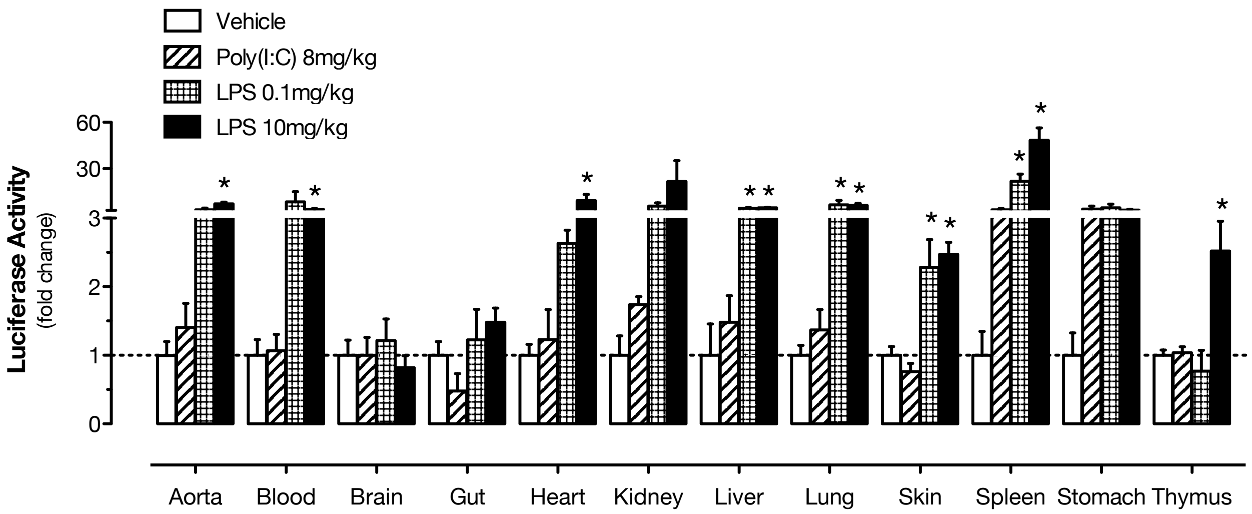

Supplementary Figure 1 Expression from the *Cox2* gene induced in mice by prototypical bacterial- and viral-type PAMPs, measured as luciferase activity in tissue homogenates

Cox2 promoter-driven luciferase activity was measured in tissue from *Cox2*$^{fLuc/+}$ luciferase reporter mice by luminescent emission, in the presence of saturating concentration of D-luciferin, from tissue homogenates. As observed with bioluminescent imaging (Figure 1), the bacterial-type PAMP, LPS, produced a dose-dependent induction of *Cox2* gene-driven luciferase expression across a broad range of tissues. The viral-type PAMP, poly(I:C), however, only produced a detectable up regulation of *Cox2* gene driven luciferase expression in the spleen and stomach. Data are expressed as means ± s.e.m from n=4-5 individual animals per treatment. *, $p<0.05$ vs. vehicle.

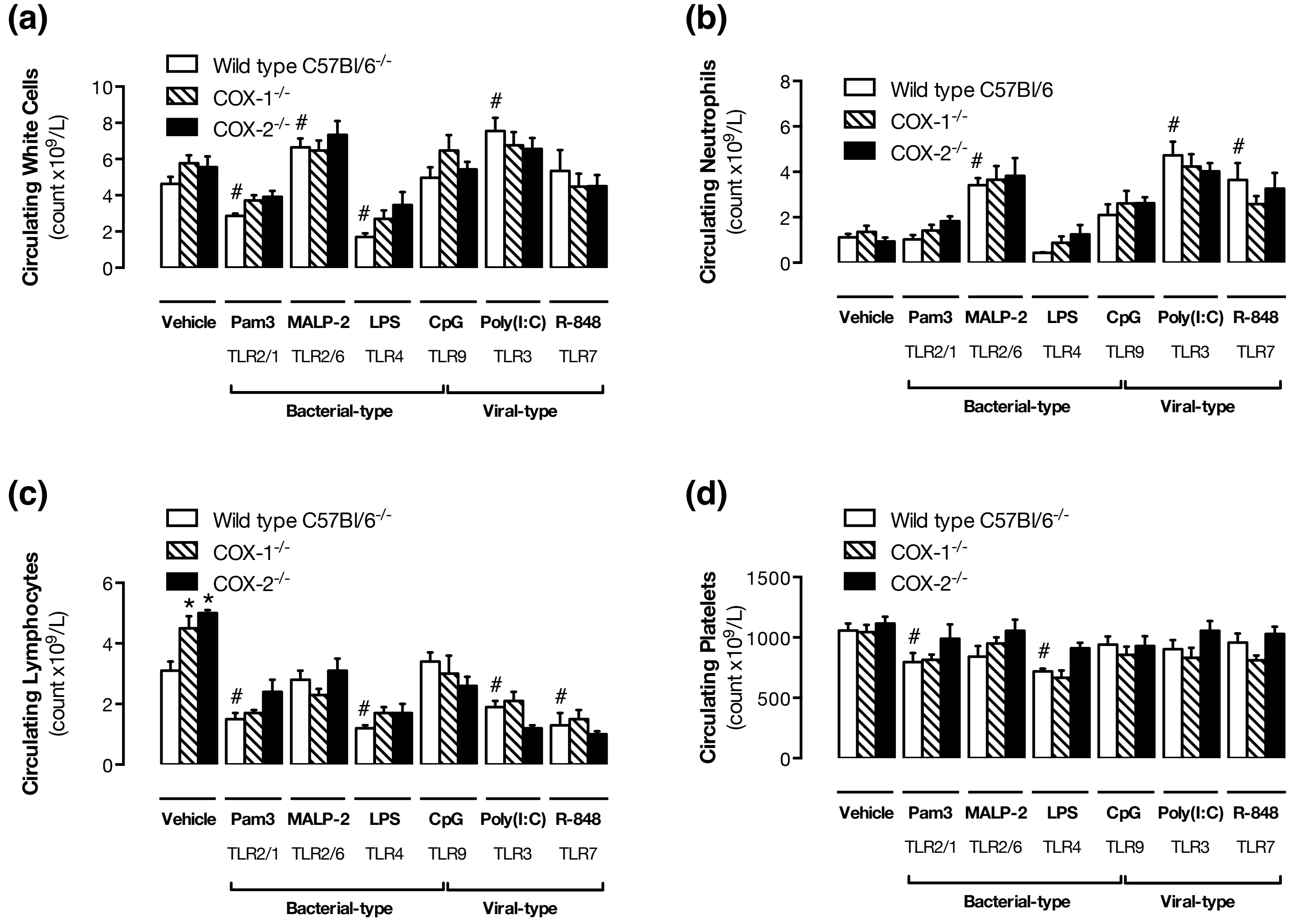

Supplementary Figure 2 Effect of *Cox1* and *Cox2* gene deletion on circulating blood cell counts in mice treated with bacterial and viral PAMPs

Wild-type, *Cox1*$^{-/-}$ and *Cox2*$^{-/-}$ mice were treated with a range of bacterial and viral PAMPs. After 4 hours circulating counts of total while cells (a), neutrophils (b), lymphocytes (c) and platelets (d) were measured. Although treatment with PAMPs produced distinct patterns of changes in circulating cell counts, none of these responses were altered by *Cox1* or *Cox2* gene deletion. Data are expressed as means ± s.e.m from n=6-12 individual animals per treatment. *, $p<0.05$ vs. wild-type; $^{\#}$, $p<0.05$ vs. vehicle-treated wild-type.

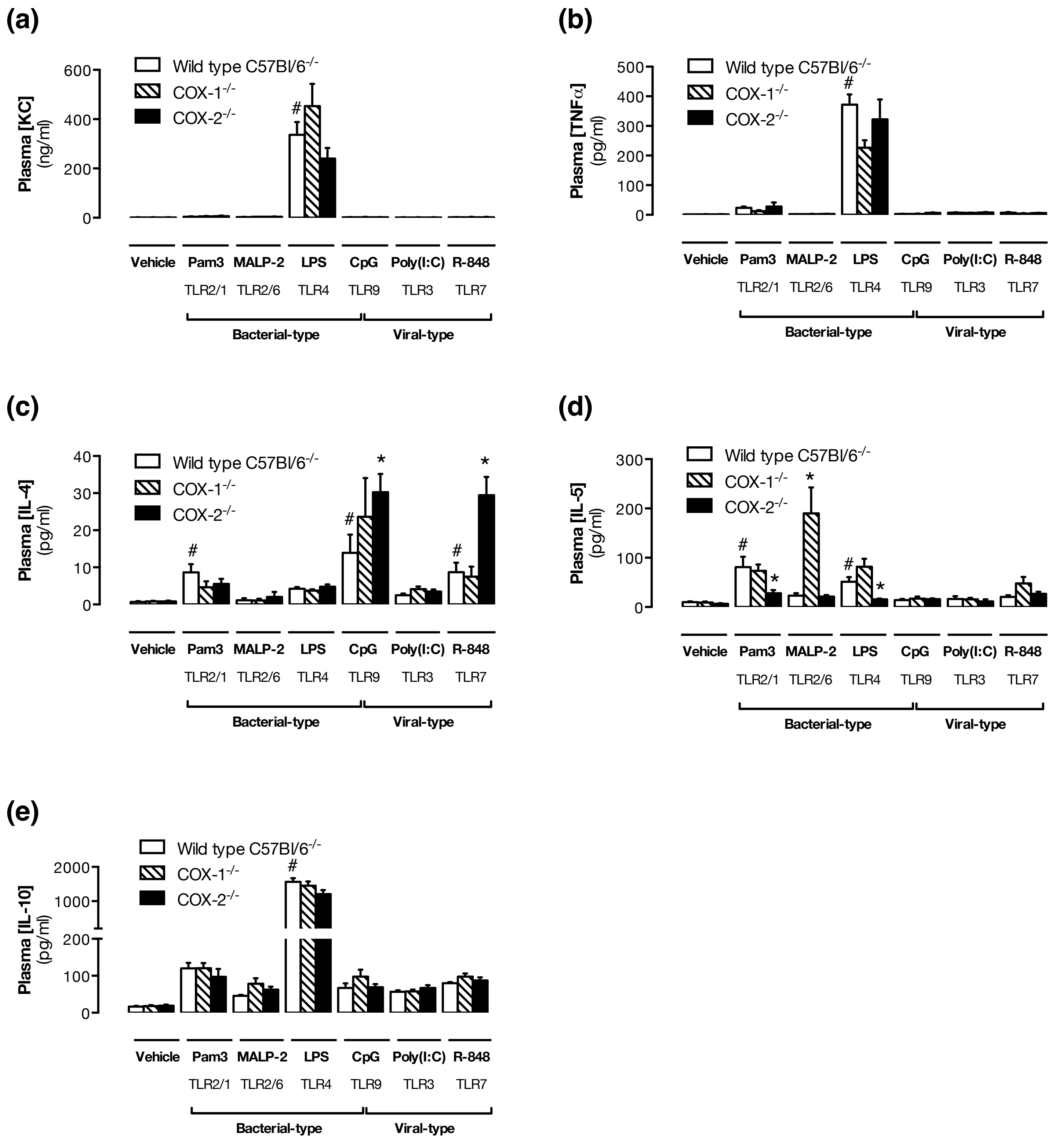

Supplementary Figure 3 Effect of *Cox1* and *Cox2* gene deletion on circulating pro- and anti-inflammatory cytokine levels in mice treated with bacterial and viral PAMPs

Wild-type, *Cox1*$^{-/-}$ and *Cox2*$^{-/-}$ mice were treated with a range of bacterial and viral PAMPs. After 4 hours, the levels of KC (a), TNFα (b), IL-4 (c), IL-5 (d) and IL-10 (e) were measured. Plasma KC, TNFα and IL-10 levels were increased by LPS but not other PAMPs. Plasma levels of IL-4 were increased by Pam3CSK4, CpG ODN and R-848. Plasma levels of IL-5 were increased Pam3CSK4, MALP-2, LPS and R-848. *Cox2* gene deletion reduced the IL-5 response to LPS and Pam3CSK4, and boosted the IL-4-response to CpG ODN and R-848. *Cox1* gene deletion enhanced the IL-5 response to MALP2. Data are expressed as means ± s.e.m from n=6-12 individual animals per treatment. *, $p<0.05$ vs. wild-type; #, $p<0.05$ vs. vehicle-treated wild-type.

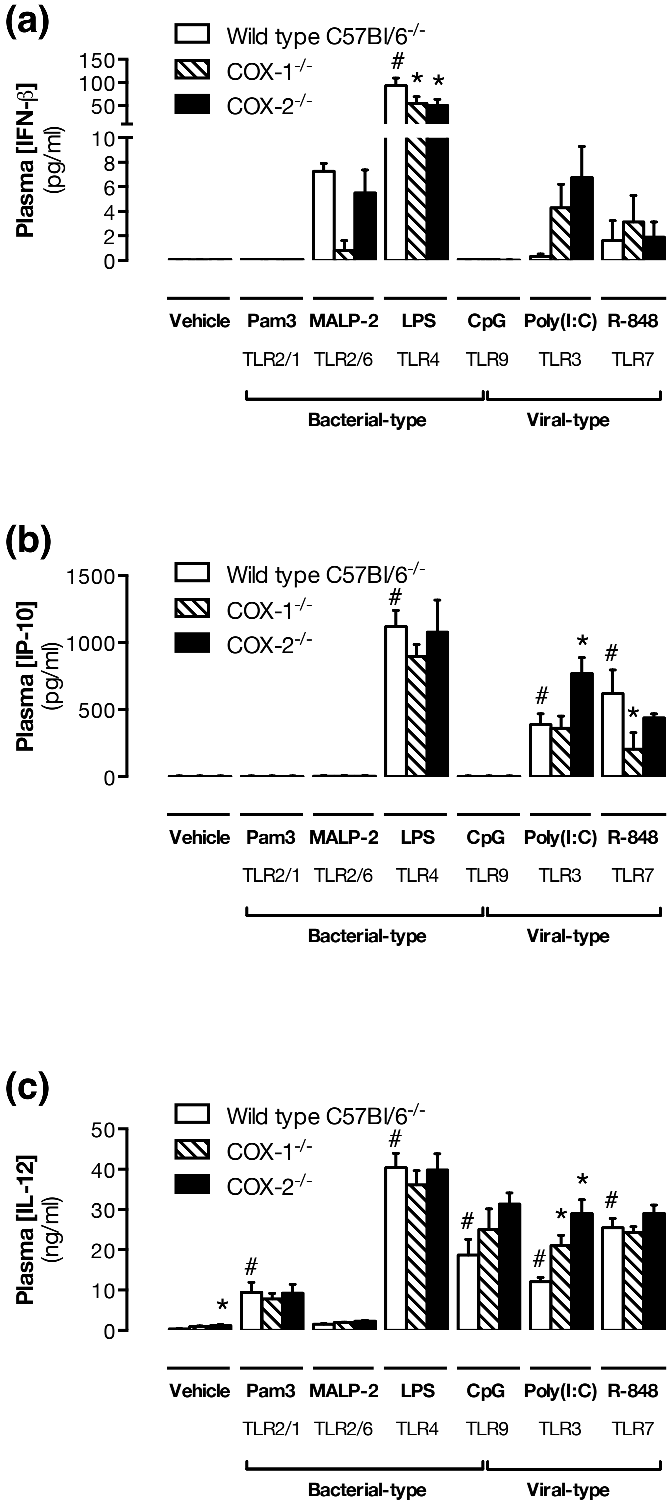

Supplementary Figure 4 Effect of *Cox1* and *Cox2* gene deletion on circulating levels of interferons and interferon-related cytokines in mice treated with bacterial- and viral-type PAMPs

Wild-type, *Cox1*$^{-/-}$ and *Cox2*$^{-/-}$ mice were treated with a range of bacterial- and viral-type PAMPs. After 4 hours, levels of IFNβ (a), IP-10 (b) and IL-12 (c) were measured. Plasma IFNβ levels were significantly increased in wild-type mice only by LPS. Plasma IP-10 levels were elevated by treatment with poly(I:C), LPS and R-848. Plasma IL-12 levels were increase by all stimuli except MALP-2. *Cox2* deletion enhanced basal IL-12 levels, the IL-12 response to CpG ODN and the IFNβ, and IL-12 and IP-10 responses to poly(I:C). *Cox1* deletion also enhanced the IL-12 response to poly(I:C), but supressed the IP-10 response to R-848. Data are expressed as means ± s.e.m from n=6-12 individual animals per treatment. *, $p<0.05$ vs. wild-type; #, $p<0.05$ vs. vehicle-treated wild-type.

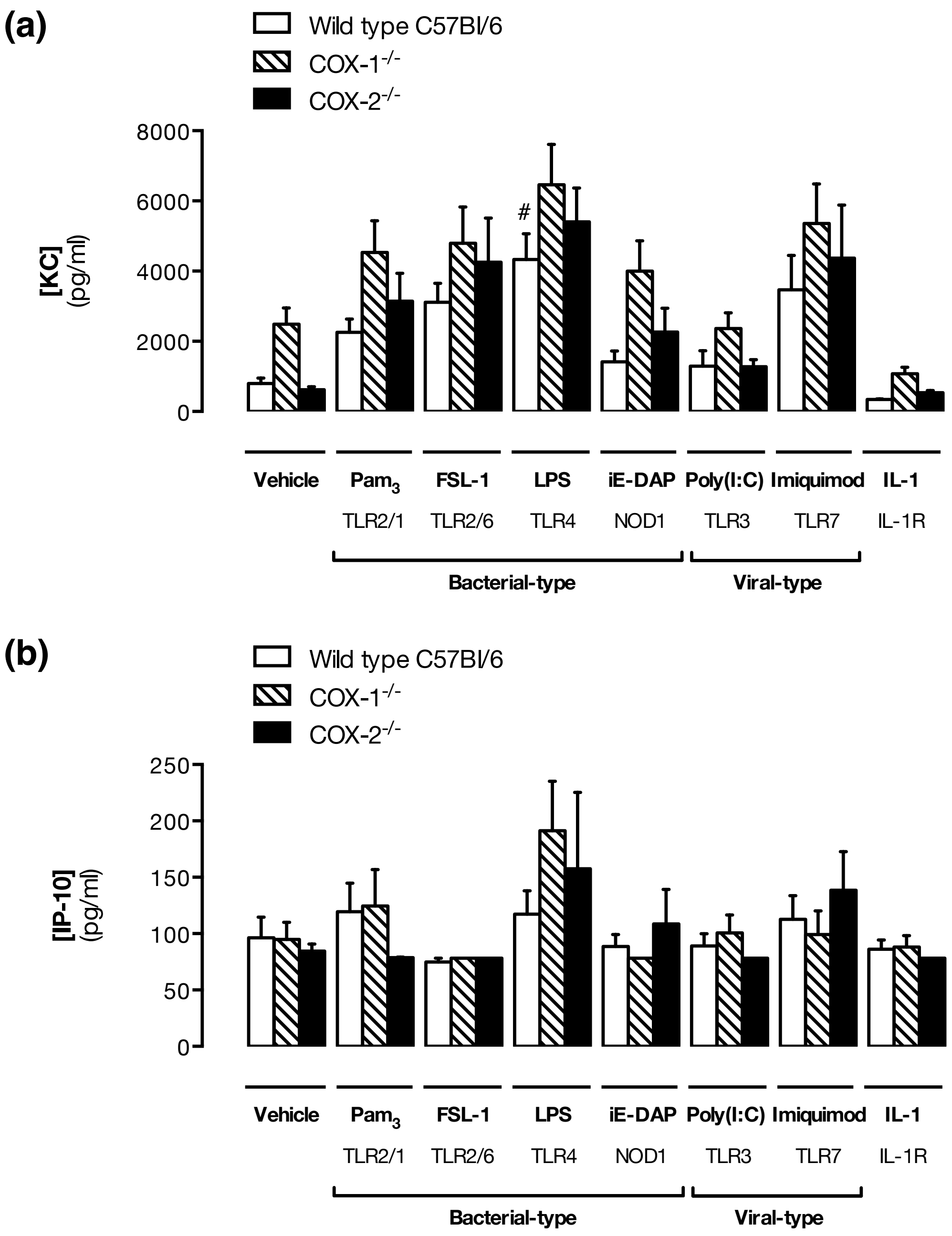

Supplementary Figure 5 Effect of *Cox1* and *Cox2* gene deletion on cytokine levels in blood treated with bacterial and viral PAMPs in culture.

Blood was collected from wild-type, *Cox1*$^{-/-}$ and *Cox2*$^{-/-}$ mice and treated in culture with a range of bacterial and viral PAMPs. After 24 hours levels of the pro-inflammatory cytokine KC (a) and the interferon-response cytokine IP-10 (b) were measured in plasma. In this ex vivo assay, only LPS increased KC levels, whilst no tested PAMP altered IP-10 levels. *Cox1* or *Cox2* gene deletion had no effect on levels of either cytokine in response to any tested PAMP. Data are expressed as means ± s.e.m from n=8 individual animals per treatment. *, $p<0.05$ vs. wild-type; $^{\#}$, $p<0.05$ vs. vehicle-treated wild-type.
